# Effects of Lightning on the Magnetic Properties of Volcanic Ash

**DOI:** 10.1038/s41598-019-41265-3

**Published:** 2019-03-18

**Authors:** Kimberly Genareau, Yang-Ki Hong, Woncheol Lee, Minyeong Choi, Mojtaba Rostaghi-Chalaki, Pedram Gharghabi, James Gafford, Joni Klüss

**Affiliations:** 1 Department of Geological Sciences, The University of Alabama, Tuscaloosa, Alabama 35487 USA; 2 Department of Electrical and Computer Engineering, The University of Alabama, Tuscaloosa, Alabama 35487 USA; 3 Department of Electrical and Computer Engineering, Mississippi State University, Starkville, Mississippi 39762 USA; 4 Center for Advanced Vehicular Systems at Mississippi State University, Starkville, Mississippi 39759 USA; 5 Present Address: Energy Production and Infrastructure Center, The William States Lee College of Engineering, University of North Carolina, 9201 University City Blvd, Charlotte, North Carolina 28223 USA

## Abstract

High-current impulse experiments were performed on volcanic ash samples to determine the magnetic effects that may result from the occurrence of volcanic lightning during explosive eruptions. Pseudo-ash was manufactured through milling and sieving of eruptive deposits with different bulk compositions and mineral contents. By comparing pre- and post-experimental samples, it was found that the saturation (i.e., maximum possible) magnetization increased, and coercivity (i.e., ability to withstand demagnetization) decreased. The increase in saturation magnetization was greater for compositionally evolved samples compared to more primitive samples subjected to equivalent currents. Changes in remanent (i.e., residual) magnetization do not correlate with composition, and show wide variability. Variations in magnetic properties were generally more significant when samples were subjected to higher peak currents as higher currents affect a greater proportion of the subjected sample. The electrons introduced by the current impulse cause reduction and devolatilization of the ash grains, changing their structural, mineralogical, and magnetic properties.

## Introduction

**Figure 1:**
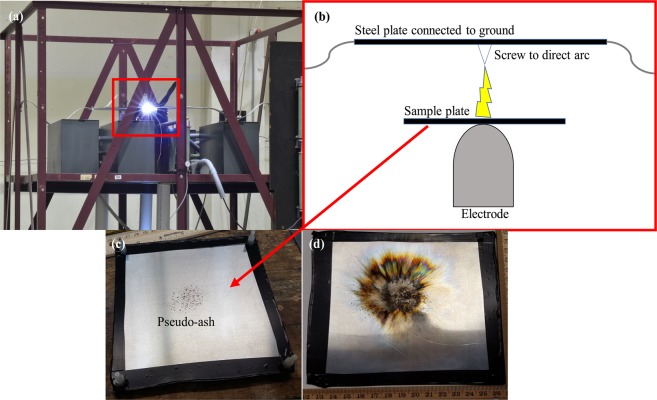
Experimental apparatus. (**a**) Faraday cage containing the capacitor bank and electrode, which supports the sample. The red box outlines where the pseudo-ash sample is located and this is further detailed in the sketch in; (**b**) the plate supporting the ash sits atop the electrode, which discharges to the steel plate above, allowing the arc to travel through the ash before reaching ground. A screw was inserted into the steel plate to direct the arc through the sample. The sample plate is attached to the steel plate with four acrylic screws not shown in this sketch (not to scale); (**c**) sample plate with pseudo-ash dusted in the center prior to the impulse experiment; (**d**) sample plate following the impulse experiment.

Lightning-induced volcanic spherules (LIVS) have been observed in ashfall deposits from explosive volcanic eruptions where lightning was documented^1^. LIVS provide an important record of electrical activity inside eruptive columns and plumes, but they are small in size (<100 μm) and difficult to find amongst unaltered ash particles. LIVS are easily distinguished from unaltered ash particles due to their spherical shape, and although the morphological effects of lightning are obvious, the effects of lightning on the magnetic properties of volcanic ash have not been constrained until now. Previous current impulse experiments^2^ revealed that ash grains are morphologically altered depending upon their distance from the axis of the discharge channel. Using current impulses with peak values of ~100 kA, it was shown that the wide range of temperatures within the channel may exceed both the Curie points and melting points of igneous minerals depending upon the distance from the channel axis. Within the center of the channel, temperatures generated are a function of the peak current and greatly exceed those required to disassociate compounds (>20,000 °C). 100 kA was initially used to represent the upper limit of potential volcanic lightning peak currents, as there have been few studies able to directly measure these values. One study^3^ measured average peak currents of 2 kA in cloud-to-ground (CG) discharges at Sakurajima volcano in Japan. Another study^4^ reported peak currents of up to ~50 kA, as recorded by the Vaisala lightning detection network, during the 2017 eruption of Bogoslof in Alaska. Peak currents from thunderstorm lightning CG flashes can range from 1 to 200 kA, with an average of 30 kA^5^, yet thunderstorm discharge events have been measured much more frequently than lightning that occurs during explosive volcanic eruptions. For the study presented here, we subject volcanic ash samples to peak currents of ~7 kA, ~25 kA, and ~100 kA using a current impulse generator (Fig. 1) to cover the possible range of volcanic lightning values.

The existence of fulgurites, products formed in rocks and sediments struck by CG events, suggest some possible chemical and magnetic effects of volcanic lightning on ash particles. Previous analyses of fulgurites found throughout the world have revealed that the geologic materials are chemically reduced as a result of lightning discharge^6–12^. This reduction has been attributed to several possible mechanisms, including electrolysis^6^, the presence of carbon as a reducing agent^6,13^, shockwave-induced reduction^14^, thermodynamic instability of particular oxides at high temperatures^6,15^, and conversion of an oxide to a more oxidized state at the expense, and consequent reduction, of a less stable oxide compound^8^. Examination of oxide compounds subjected to rocket-triggered lightning have revealed that the thermodynamic stability of the exposed compound will play a large role in whether or not the oxide is reduced to the metallic state^8^. Thus, not all oxide compounds will experience the same effects, even when subjected to equivalent lightning currents and corresponding temperatures.

The effects of lightning on geologic materials have been previously documented in a number of locations and several studies have attributed variability in the surficial magnetic characteristics of rock outcrops (a.k.a., *anomalous magnetization*) to CG lightning strikes^16–21^. These lightning-induced magnetic anomalies have posed problems for those attempting to survey regions for unexploded ordnance^22^ or archaeological sites^23,24^. These previous studies have shown that CG strikes will impart a *lightning-induced remanent magnetization* (LIRM), which alters the natural remanent magnetization (NRM), and has been used in some studies to calculate characteristics (e.g., current, polarity) of the discharge^21,25^. Using current impulse experiments, one study^26^ attributed changes in the magnetic properties of rocks to changes in mineralogy, where existing ferrimagnetic magnetite (Fe_3_O_4_) transformed to antiferromagnetic hematite (α-Fe_2_O_3_) through oxidation. In other studies of fulgurites, mineralogical changes typically assumed to result from meteorite impacts, such as shock lamellae in quartz^27,28^ have been documented. Thus, chemical and mineralogical changes can occur either thermally, due to the high temperatures within the discharge channel, or isothermally due to the strong magnetic field or high pressures imposed by the lightning strike.

The existence of LIVS in ashfall samples provides textural evidence of volcanic lightning occurrence. If ashfall samples can also provide an opportunity to quantify the characteristics of the lightning discharge, this will allow constraint of lightning parameters even if the phenomenon is not directly observed. During the transport of volcanic ash through the atmosphere, numerous lightning discharge events may occur. Each discharge is unique in terms of the peak current, temperature, duration, and channel length. Because volcanic ash is composed of different igneous minerals and oxide compounds within these minerals, in addition to those within an amorphous glass phase, lightning discharge effects on volcanic ash can be quite variable within a single sample and throughout the course of an eruption. This study shows, for the first time, that lightning will cause variations in the magnetic properties of volcanic ash and the amount of that variation will be controlled by both the peak current of the discharge and the bulk composition of the ash.

## Results

X-Ray fluorescence (XRF) analyses of the pre-experimental pseudo-ash samples reveal the relative concentrations of major and minor element oxides (Table 1). The basic samples from Lathrop Wells volcano (LW-ONW and LW-NINW) contain relatively lower amounts of SiO_2_ and higher amounts of Fe_2_O_3_ and other metal oxides (e.g., MgO, TiO_2_) compared to the intermediate (R-1, K38-PC2, SHV347-B) and acidic samples (OBS1 and ECF2LAP), which is displayed in the calculated ratio of basic to acidic compounds, *R*_*b/a*_ (Table 1). The oxide compounds measured with the XRF may be present in either the volcanic glass, or in particular mineral phases. XRD analyses provide the dominant minerals within each sample. The acidic samples contain less minerals and higher proportions of glass compared to the other samples. OBS1 contains small amounts of quartz and plagioclase (Fig. S1) while ECF2LAP also contains those minerals, in addition to titanomagnetite (Fig. S2). The basic samples contain olivine, plagioclase feldspar, and magnetite (Figs S3, S4). All the intermediate samples contain various amounts of plagioclase feldspar, magnetite, ortho-/clinopyroxene, and the Soufrière Hills sample also contains hornblende (Figs S5–S7). Instrument limitations require **>**2 volume % of a phase, so trace minerals will not be detected.

**Table 1:** Bulk compositions of pre-experimental pseudo-ash samples measured with X-ray fluorescence.

Bulk oxide abundance (wt %)	LW-NINW	LW-ONW	R-1	K38-PC2	SHV347-B	OBS1	ECF2LAP*
	Basic samples		Intermediate samples			Acidic samples	
SiO_2_	48.78	49.12	53.56	53.43	58.11	76.19	72.75
TiO_2_	1.96	1.90	0.78	0.61	0.64	0.14	0.32
Al_2_O_3_	17.13	17.14	17.98	19.39	18.21	12.71	14.19
Fe_2_O_3_	11.29	11.36	9.21	8.79	7.34	1.32	2.10
MnO	0.18	0.18	0.16	0.23	0.19	0.05	0.05
MgO	5.90	5.73	6.06	4.37	2.95	0.15	0.68
CaO	8.27	8.46	7.80	9.74	7.70	0.44	1.66
Na_2_O	3.49	3.22	2.40	2.83	3.61	4.40	3.81
K_2_O	1.82	1.73	0.99	0.56	0.79	4.59	4.35
P_2_O_5_	1.19	1.16	0.12	0.13	0.14	0.02	0.09
Total	100	100	99	100	100	100	100
R_*b/a*_	0.45	0.44	0.37	0.36	0.29	0.12	0.14

Each value is an average of five individual measurements. Based upon these values, the two basic samples (LW-NINW and LW-ONW) are classified as trachybasalts, the three intermediate samples (R-1, K38-PC2, and SHV347-B) are considered basaltic andesites/andesites, and the acidic samples (OBS1 and ECF2LAP) are classified as rhyolites. *R*_*b/a*_ is the ratio of basic to acidic oxides and is calculated as *R*_*b/a*_ = (CaO + Fe_2_O_3_ + MgO + K_2_O + Na_2_O + MnO)/(SiO_2_ + Al_2_O_3_ + TiO_2_ + P_2_O_5_) following previous methods^32^. *Values for ECF2LAP are not those of the exact sample used in the experiments, but for a different pyroclast derived from the same eruptive sequence.

**Figure 2:**
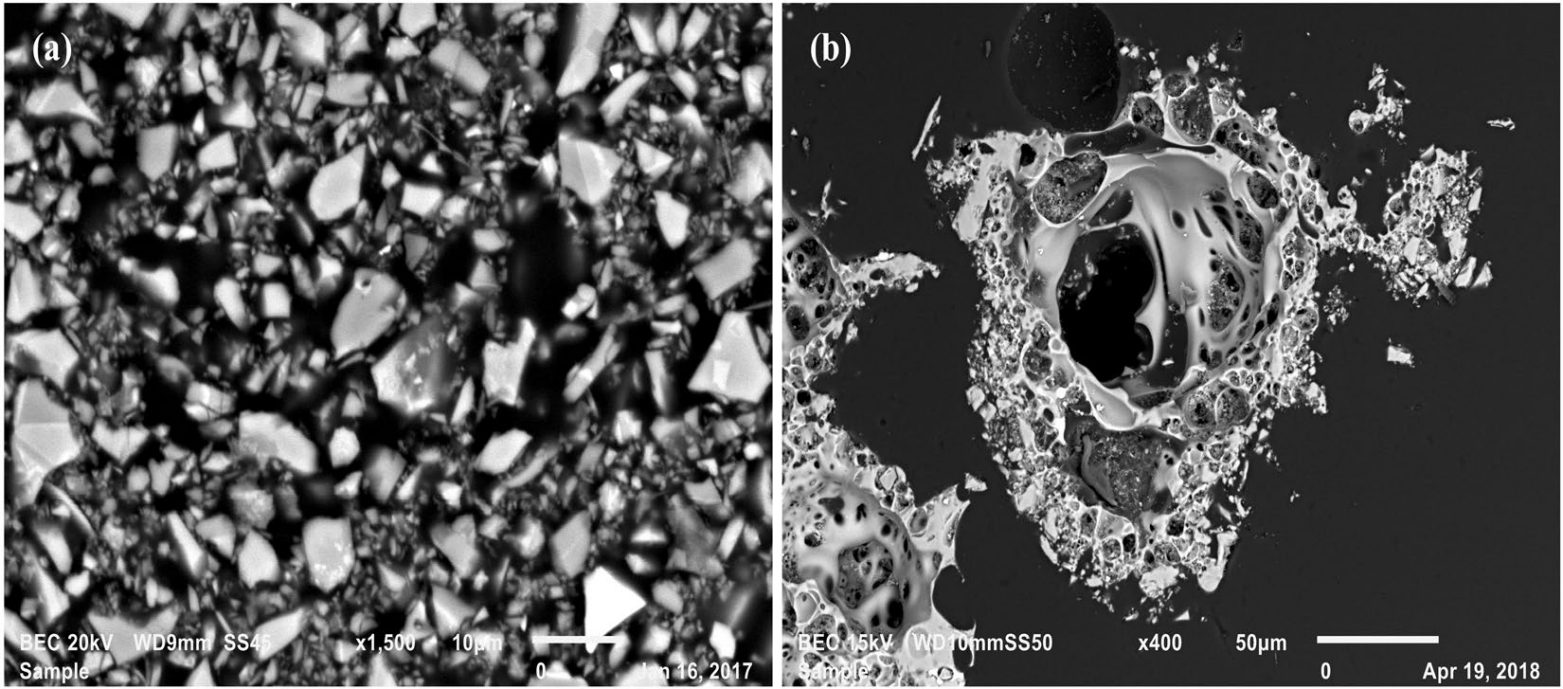
Scanning electron microscope images of pseudo-ash samples. (**a**) Backscattered electron image of polished epoxy mount of sample LW-ONW prior to current impulse experiments. All grains are <32 μm in size and have a consistent aspect ratio of ~0.5. (**b**) Backscattered electron image of post-experimental sample. For all post-experimental samples, aggregates of individual grains melted together and vesiculated, forming foamed particles **>**100 μm in diameter.

**Figure 3:**
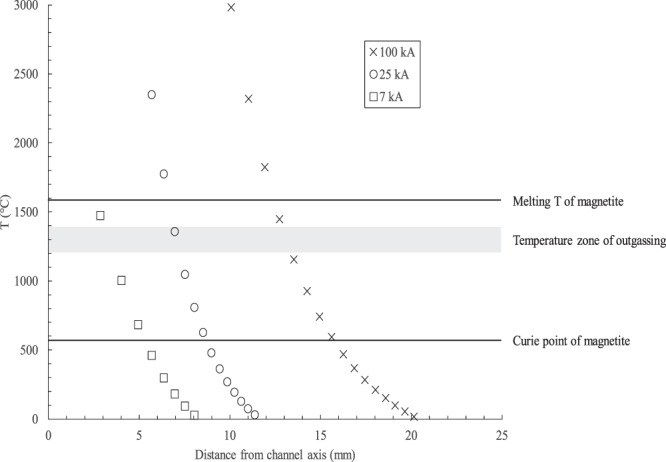
Temperature within the discharge channel as a function of peak impulse current. The grey rectangle indicates the zone where particles will undergo degassing, forming bubbles from the escape of volatiles, as determined through sintering experiments on volcanic ash^29^. For reference, the upper line shows the melting temperature (~1590 °C) and the lower line shows the Curie point (~575 °C), respectively, of the mineral magnetite. Volcanic ash located within this region of the discharge channel may undergo magnetic changes without any structural modification to magnetite grains, posing important implications for the use of this mineral as a paleomagnetic indicator in distal ashfall samples.

For all post-experimental samples subjected to a peak current of ~100 kA, scanning electron microscope (SEM) secondary electron images reveal aggregates of individual grains melted together and vesiculated (Fig. 2), indicating volatilization of residual H_2_O and CO_2_ or deoxygenation of oxide compounds. The high temperatures generated by the current impulse can reach above the melting point of the ash, so any particles within the appropriate temperature zone of the discharge channel will be morphologically altered, and some will be vaporized^2^. The outgassing of these particle aggregates corresponds to temperatures between 1200 and 1400 °C based on sintering experiments conducted on intermediate volcanic ash samples^29^. Using previous equations^2^ to calculate the temperature as a function of distance from the arc channel axis, grains located between 13 and 14 mm away from the axis will fall within this temperature range when subjected to a peak current of ~100 kA. For a peak current of ~25 kA, this zone exists between 7 and 8 mm from the channel axis, and for a peak current of ~7 kA, this zone is between 3 and 4 mm. This zone of outgassing is approximately 1 mm regardless of peak current (Fig. 3).

**Figure 4:**
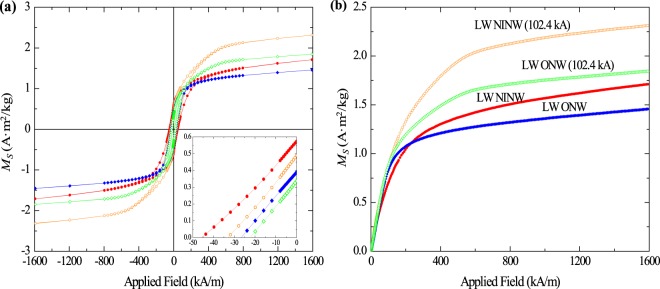
Variations in magnetization (M_s_) between pre-experimental and post-experimental basic samples as a function of applied field. (**a**) The entire loop under an applied field of −1600 to 1600 kA/m; (**b**) the positive quadrant under an applied field of 0 to 1600 kA/m. Pre-experimental hysteresis loops are labeled with the name of the sample, while post-experimental loops are labeled with the sample name followed by the peak current value that those samples were exposed to in parentheses. Error is within the symbol size.

**Figure 5:**
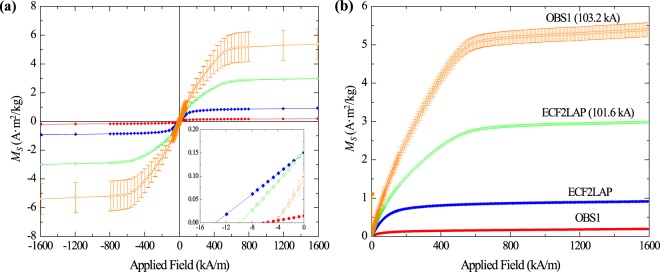
Variations in magnetization (*M*_*S*_) between pre-experimental and post-experimental acidic samples as a function of applied field. (**a**) The entire loop under an applied field of −1600 to 1600 kA/m; (**b**) the positive quadrant under an applied field of 0 to 1600 kA/m. Pre-experimental hysteresis loops are labeled with the name of the sample, while post-experimental loops are labeled with the sample name followed by the peak current value in parentheses. Error is within the symbol size. The small amount of OBS1 available for analysis following the current impulse experiments introduced greater uncertainty into the post-experimental measurements, which is why the error bars are much larger.

**Figure 6:**
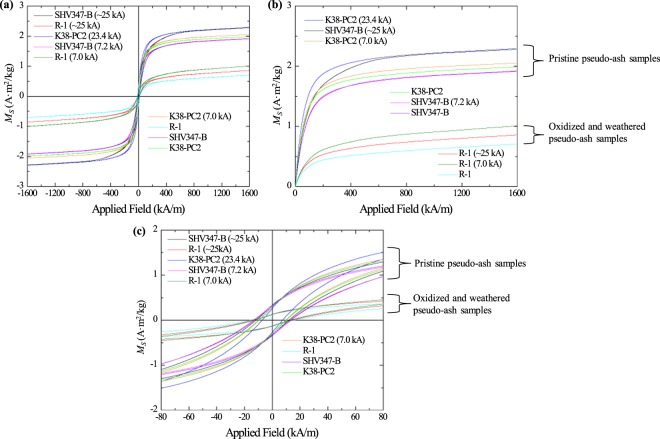
Variations in magnetization (*M*_*S*_) between pre-experimental and post-experimental intermediate samples as a function of applied field. Pre-experimental hysteresis loops are labeled with the name of the sample, while post-experimental loops are labeled with the sample name followed by the peak current value in parentheses. Pristine samples (K38-PC2 and SHV347-B) have higher magnetization values than sample R-1, which is much older and was weathered and oxidized prior to collection. (**a**) The entire loop under an applied field of −1600 to 1600 kA/m; (**b**) the positive quadrant under an applied field of 0 to 1600 kA/m; (**c**) the entire loop under an applied field of −80 to 80 kA/m. Error is within the thickness of the curve. The pristine, unaltered pseudo-ash samples are differentiated from the oxidized and weathered pseudo-ash samples due to their higher magnetization.

Vibrating sample magnetometry (VSM) was used to measure the saturation magnetization (*M*_*s*_), the remanent magnetization (*M*_*r*_), and the coercivity (*H*_*c*_) of the pre-experimental pseudo-ash. VSM analyses of all pre- and post-experimental samples can be found in Table 2, and the percent change in the different values, to provide easier comparison, can be found in Table 3. Two different sets of the intermediate samples were measured with the VSM to check for instrument and sample consistency. VSM measurements reveal that *M*_*s*_ increased for all samples exposed to a peak impulse current of ~100 kA. The increase in the basic samples was 27% and 36% for LW-ONW and LW-NINW (Fig. 4), respectively, while the increase for the acidic samples was 2595% and 224% for OBS1 and ECF2LAP, respectively (Fig. 5; Table 3). Due to limited sample quantity, the error in the OBS1 measurements were larger than those for the other samples, so the high percentage increase in *M*_*s*_ should be cautiously considered. *M*_*r*_ *decreased* for the basic samples (13–14%) but *increased* (7–350%) for the acidic samples. *H*_*c*_ decreased for both sets of samples. This decrease is slightly greater for the acidic samples (28–31%) compared to the basic samples (16–27%), but pre-experimental *H*_*c*_ values were initially lower in the former compared to the latter (Table 2). The three intermediate pseudo-ash samples were subjected to impulse currents with peak values of ~25 kA and ~7 kA (Fig. 6). Two of these samples (SHV347-B and K38-PC2) were derived from pristine volcanic deposits erupted in recent years while the third (R-1) was collected thousands of years after initial deposition and was subsequently oxidized and weathered. This post-depositional alteration is revealed in the VSM analyses and resulting hysteresis loops (Fig. 6). The two pristine samples show an increased *M*_*s*_ of 1–3% and 15–19% for a peak current of ~7 kA and ~25 kA, respectively. The decrease in *M*_*r*_ for these samples is 0–6% and 6–10% for a peak current of ~7 kA and ~25 kA, respectively. The decrease in *H*_*c*_ is 2–6% and 12–26% for a peak current of ~7 kA and ~25 kA, respectively (Table 3). An analysis of a second set of post-experimental intermediate pseudo-ash samples show some similar changes: The two pristine samples show an increased *M*_*s*_ of 6–7% and 16–29% for a peak current of ~7 kA and ~25 kA, respectively and a decreased *H*_*c*_ of 5–20% and 11–19% for a peak current of ~7 kA and ~25 kA, respectively (Table 3). There is typically a greater change in magnetic properties for the higher impulse current. However, for the older, oxidized sample (R-1), variations are greater for the lower peak current compared to the higher current. Considering that lightning is more often observed during explosive eruptions of volcanoes with intermediate to acidic magma compositions^30^, relationships between peak impulse current and changes in magnetic properties for the acidic and (pristine) intermediate pseudo-ash samples are the most relevant for the purposes of this study.

**Table 2:** Vibrating sample magnetometer measurements.

	LW-ONW	LW-ONW (102.4 kA)	LW-NINW	LW-NINW (102.4 kA)	OBS1	OBS1 (103.2 kA)	ECF2LAP	ECF2LAP (101.6 kA)	R-1	R-1 (~25 kA)	R-1 (7.0 kA)	K38-PC2	K38-PC2 (23.4 kA)	K38-PC2 (7.0 kA)	SHV347-B	SHV347-B (~25 kA)	SHV347-B (7.2 kA)
*M*_s_ (A·m^2^/kg)	1.46	1.85	1.71	2.32	0.20	5.39	0.92	2.98	0.71	0.86	1.01	1.99	2.29	2.05	1.91	2.28	1.92
*M*_*r*_ (A·m^2^/kg)	0.39	0.34	0.57	0.49	0.02	0.09	0.15	0.16	0.13	0.13	0.12	0.30	0.27	0.30	0.34	0.32	0.32
*H*_*c*_ (kA/m)	26.58	22.36	45.68	33.18	6.37	4.38	13.53	9.71	15.68	14.16	12.97	9.87	7.32	9.63	13.45	11.86	12.65
									**R-1**	**R-1 (25.4 kA)**	**R-1 (7.2 kA)**	**K38-PC2**	**K38-PC2 (25.6 kA)**	**K38-PC2 (6.9 kA)**	**SHV347-B**	**SHV347-B (24.9 kA)**	**SHV347-B (7.0 kA)**
*M*_*s*_ (A·m^2^/kg)									0.67	1.70	2.04	1.99	2.31	2.10	1.91	2.47	2.05
*M*_*r*_ (A·m^2^/kg)									0.12	0.13	0.15	0.30	0.30	0.29	0.34	0.35	0.30
*H*_*c*_ (kA/m)									15.28	9.71	6.45	10.27	8.28	9.71	13.29	11.86	10.58

Measurement of all samples in the upper half of the table, with pre-experimental values labeled with just the sample name and post-experimental values labeled with the sample name and the peak current value in parentheses; Repeated measurement of intermediate samples in the lower half of the table to check for pre-experimental consistency and post-experimental variability. Saturation magnetization (*M*_*s*_), remanent magnetization (*M*_*r*_), and coercivity (*H*_*c*_) are shown.

**Table 3:** Percent change in magnetic properties as a function of peak current.

	LW-ONW (102.4 kA)	LW-NINW (102.4 kA)	OBS1 (103.2 kA)	ECF2LAP (101.6 kA)	R-1 (~25 kA)	R-1 (7.0 kA)	K38-PC2 (23.4 kA)	K38-PC2 (7.0 kA)	SHV347-B (~25 kA)	SHV347-B (7.2 kA)
*M*_s_ (A·m^2^/kg)	↑27%	↑36%	↑2595%	↑224%	↑21%	↑42%	↑15%	↑3%	↑19%	↑1%
*M*_*r*_ (A·m^2^/kg)	↓13%	↓14%	↑350%	↑7%	0%	↓8%	↓10%	0%	↓6%	↓6%
*H*_*c*_ (kA/m)	↓16%	↓27%	↓31%	↓28%	↓10%	↓17%	↓26%	↓2%	↓12%	↓6%
					**R-1 (25.4** **kA)**	**R-1 (7.2** **kA)**	**K38-PC2 (25.6** **kA)**	**K38-PC2 (6.9** **kA)**	**SHV347-B (24.9** **kA)**	**SHV347-B (7.0** **kA)**
*M*_*s*_ (A·m^2^/kg)					↑153%	↑204%	↑16%	↑6%	↑29%	↑7%
*M*_*r*_ (A·m^2^/kg)					↑8%	↑25%	0%	↓3%	↑3%	↓12%
*H*_*c*_ (kA/m)					↓36%	↓58%	↓19%	↓5%	↓11%	↓20%

Measurement of all samples in upper half of the table; Repeated measurement of intermediate samples in the lower half of the table to check for pre-experimental consistency and post-experimental variability. Saturation magnetization (*M*_*s*_) increases for all samples, but the increase is more significant for the acidic pseudo-ash (OBS1 and ECF2LAP) compared to the basic samples (LW-NINW and LW-ONW) and intermediate samples (R-1, K38-PC2, SHV347-B). Remanent magnetization (*M*_*r*_) decreases for the basic samples, increases for the acidic samples, and both increases and decreases for the intermediate samples. Coercivity (*H*_*c*_) decreases for all samples. Percent change in values is generally greater for more compositionally evolved samples and for those subjected to higher peak currents.

**Figure 7:**
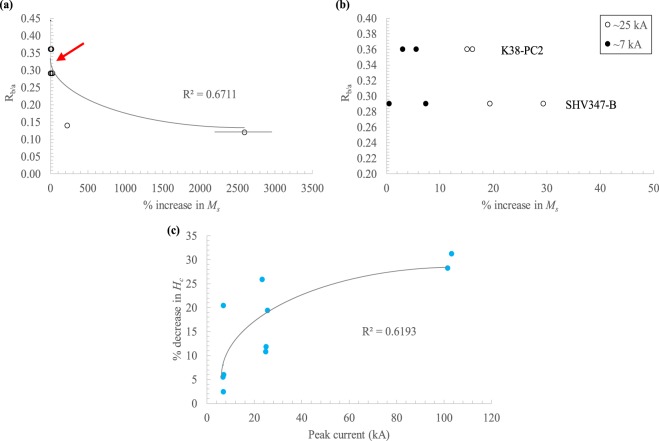
Correlations between changes in magnetic properties and experimental conditions. (**a**) Pseudo-ash samples with a lower ratio of basic to acidic oxide compounds (*R*_*b/a*_) show a higher increase in the saturation magnetization (*M*_*s*_) for the pristine intermediate and acidic pseudo-ash, with red arrow indicating; (**b**) the intermediate samples, showing that there is generally a greater increase in *M*_*s*_ for samples with a lower *R*_*b/a*_ and also those exposed to a higher peak current; (**c**) Pristine intermediate and acidic pseudo-ash show a positive correlation between the peak current of the impulse and the % decrease in coercivity (*H*_*c*_).

Potential relationships between experimental results and pseudo-ash properties are provided in Fig. 7. There is a moderate power law correlation between the *R*_*b/a*_ and the increase in *M*_*s*_ for the acidic and pristine intermediate samples, with lower *R*_*b/a*_ values corresponding to a greater increase in magnetization (Fig. 7a). When only the two pristine intermediate samples (K38-PC2 and SHV347-B) are plotted, this relationship disappears, but there is still a noticeably higher % increase in *M*_*s*_ for both the samples with lower *R*_*b/a*_ and also the samples exposed to a higher peak current (Fig. 7b). Additionally, there is a moderate logarithmic correlation between the decrease in *H*_*c*_ and the peak current of the impulse for the acidic and pristine intermediate samples (Fig. 7c).

## Discussion

The portion of sample available for analyses following the current impulse experiments is always lower than the amount of starting material due to destruction or loss of some portion^2^. Although we start with 1–2 grams of pseudo-ash, the recovered portion following the experiments is approximately 25% for 100 kA impulses, 50–75% for 25 kA impulses, and 75–90% for 7 kA impulses. More of the acidic samples were destroyed or lost during the ~100 kA current impulse compared to the basic samples. Each sample was subjected to two or three different impulse experiments to ensure that enough material would survive for the variety of analyses performed, and one of each of these was used for subsequent VSM analysis, except for the intermediate samples, where two sets were analyzed. Hysteresis loops for the second set of intermediate samples can be found in Fig. S8. During initial preparation of the post-experimental samples, it was found that the majority of each sample had been magnetized. Approximately 50% of ECF2LAP was removed from the post-experimental sample with an ordinary magnet. The percentage was even greater (~75%) for LW-ONW. The other two samples subjected to a current impulse of ~100 kA were similar, with most of the post-experimental sample easily removed with a magnet. Sample masses were too small for resolution of the laboratory balance, so these percentages are visual estimates. Samples subjected to lower peak currents (i.e., intermediates) were not tested with a magnet prior to VSM analyses.

The increase in *M*_*s*_ for the pseudo-ash likely stems from an overall reduction of the oxide compounds in the bulk sample. As mentioned previously, reduction/oxidation of particular compounds will be controlled by not only the thermodynamic conditions of the environment, but also the thermodynamic stability of the oxide. The pre-experimental *M*_*s*_ of the bulk pseudo-ash sample is a function of the chemical composition and mineralogy, and any alteration of the composition or mineralogy will, consequently, alter the *M*_*s*_. For example, oxidation will convert ferrimagnetic magnetite (Fe_3_O_4_) into ferrimagnetic maghemite (γ-Fe_2_O_3_), and this results in a change of the *M*_*s*_ from 92–100 A·m^2^/kg to 60–80 A·m^2^/kg, respectively, despite the fact that both minerals are ferrimagnetic^31^. If oxidation decreases *M*_*s*_, then it appears that the reduction induced by the current impulse has caused the increase in *M*_*s*_ for the pseudo-ash samples used here. The majority of samples contain the mineral magnetite in low abundance (<10%). All samples contain plagioclase feldspar, a Ca/Na aluminosilicate, which is a dominant phase in the intermediate samples. In addition to these minerals, others present in the samples (e.g., quartz, ortho-/clinopyroxene, hornblende) all contain oxygen in the crystallographic structure. The addition of electrons to the samples disassociates this oxygen from the compounds, textural evidence of which is potentially seen in Fig. 2, reducing the pseudo-ash and increasing the *M*_*s*_. The change in grain size due to fusing of particles may also play a role.

The *R*_*b/a*_ ratio has been used in previous experiments to determine the effect of ash composition on sintering behavior, which is relevant to aviation hazards^32^. Here, there appears to be a relationship between this ratio and the amount of magnetic alteration to the pseudo-ash samples (Fig. 7a,b). The increase in *M*_*s*_ is more significant for the acidic samples, and this may be due to the lower overall melting temperatures of these samples, higher amorphous glass content, and the lower pre-experimental *M*_*s*_. The acidic samples show a greater effect because they are more susceptible to structural and chemical alteration at the high temperatures generated and are initially more oxidized. The acidic pseudo-ash samples were prepared from deposits with very low mineral content, as OBS1 was originally an obsidian lava and ECF2LAP was a crystal-poor pumice clast. These two samples, dominated by an amorphous component, will undergo changes much more rapidly when compared to the other samples with higher crystal contents. Despite the larger error in the values for OBS1, an increase or decrease in the measured *M*_*s*_ will produce a similar trend as observed in Fig. 7a. Even samples with very similar bulk compositions do not show the same percent change in the examined magnetic properties when subjected to identical impulse currents. For example, the two basic samples show an increase in *M*_*s*_ that differs by 9% and a decrease in *H*_*c*_ that differs by 11% (Table 3), despite nearly equivalent bulk chemistries (Table 1). Variations in results may stem from small differences in chemical composition of the ash, mineralogy, amount of sample directly affected, or the unique path of the arc through the samples, explaining the variability in percent change between two portions of the same intermediate sample subjected to the same peak current (Table 3). Consistency in the pre-experimental values of the intermediate samples confirms that the variations are not an analytical artifact. Because this study utilized bulk ash samples, post-experimental measurements are highly variable and thus, cannot provide a direct quantification of lightning current. However, examining the magnetic changes induced by lightning on individual ash components (e.g., minerals like magnetite or glass shards) is a fruitful topic for future study and will likely provide more quantitative information.

The experiments presented here do not reflect actual conditions during explosive volcanic eruptions and are conducted to specifically determine the magnetic effects on exposed ash grains. During eruptions, volcanic ash will be moving through a high-temperature mixture of volatiles and other particles. Because the experimental apparatus does not permit us to aerosolize the pseudo-ash samples, they must be supported on the Al alloy plate used here. Examination of both the post-experimental pseudo-ash samples and Al plates show the potential contamination by the Al plates for the 100 kA experiments. SEM images reveal very few spherules of Al alloy (~5) in all of the post-experimental pseudo-ash, which can be easily differentiated based upon texture (Fig. S9), as portions of the plate were more difficult to remove from the solid surface compared to easy mobilization of the loose ash grains. Post-experimental XRD analysis of only one sample (OBS1) revealed a peak in the spectra indicative of Al metal (Fig. S10). This peak was not observed in the XRD spectra of the other three pseudo-ash samples subjected to a current impulse of ~100 kA (Figs S11–S13). The reduction for the entire sample is an overall average caused by the total number of oxidation and reduction reactions that occurred simultaneously, which will vary according to the different oxide compounds present and may partially cancel each other out. As mentioned previously, lower current values affect lower amounts of the pseudo-ash sample, so it appears that differences between the increase in *M*_*s*_ may simply stem from the different proportions of the pseudo-ash sample that were affected. It is unlikely that the Al alloy plate contributed to the increased magnetization of the pseudo-ash, as it is in its most reduced state as a metal, but it is possible that oxidation of the alloy may have decreased the measured *M*_*s*_ value for the ~100 kA experiments (Fig. S14).

Volcanic lightning that occurs in the eruptive column or plume will only affect the ash within the discharge channel, and this ash will either fall out immediately, or be transported through the atmosphere before it is finally deposited amongst other unaltered ash grains^1^. Results shown here indicate that the magnetic properties of a bulk ash sample may show wide variations in changes to the *M*_*s*_, *M*_*r*_, and *H*_*c*_ based on the quantity that is directly affected. However, effects to individual mineral grains may be more relevant if these grains are utilized for further studies following ash deposition. Many products of explosive volcanic eruptions contain the mineral magnetite, like the majority of samples used here. Magnetite may be present as a minor mineral in magmas across a range of compositions, from basic to acidic. Magnetite can function as a paleomagnetic indicator in tephra deposits to constrain eruptive ages or the magnetic properties of ashfall layers in various environments, such as deep-sea sediments^33,34^. Since the temperatures within the lightning discharge channel can range over several orders of magnitude in a limited radius, some particles may be exposed to temperatures below their melting point but above their Curie point. The Curie temperature and melting temperature of magnetite is ~575 °C and ~1590 °C, respectively. Again, using previous equations^2^, grains located between 13 and 16 mm away from the discharge channel axis will fall within this temperature range when subjected to a peak current of ~100 kA (Fig. 3). Thus, any magnetite located within this 3 mm zone may undergo a LIRM without showing any morphologic changes. For lower peak currents, this zone is smaller: roughly 2 mm for a peak current of both 25 kA and 7 kA (Fig. 3). Thus, LIRM should be considered as a possible cause for variations in *M*_*r*_ between magnetite grains from the same ashfall layer.

Increased magnetization of volcanic ash in the atmosphere may pose an additional hazard to aircraft. Many studies have focused on the effects of volcanic ash to the operation and function of jet engine components^29,35–39^. Due to the high temperatures generated in the jet (1200–2000 °C), volcanic ash can fuse to internal regions and cause catastrophic engine failure^40–43^. The results of the study presented here indicate that lightning will increase the magnetization of volcanic ash particles, which may allow them to adhere more easily to jet engine components containing ferrous materials and alloys. However, ash that does retain an increased magnetization following lightning discharge will probably be of such limited quantity that an increased hazard to aircraft will not be statistically significant. Additionally, any ash ingested by a jet engine during flight will fuse to turbine components due to the high temperatures regardless of the increased magnetization. The relationship between ash magnetic properties and fusion behavior is beyond the scope of the present study but may be relevant for aircraft hazard assessment and is an interesting topic for future research.

In conclusion, this study shows, for the first time, that lightning will induce a change in the magnetic properties of volcanic ash grains, and the magnitude of this change will be controlled by the peak current of the discharge and the bulk composition of the ash particles, both of which will determine the proportion of ash affected. When subjected to a current impulse of either ~100 kA, ~25 kA, or ~7 kA, the volcanic ash is reduced, causing an increase in the saturation magnetization, and a decrease in the coercivity. Remanent magnetization also varied for most samples, suggesting that volcanic lightning can influence the utility of ashfall for paleomagnetic studies.

## Methods

Current impulse experiments were conducted following previous methods^2^ on seven different ash samples using the experimental facilities at Mississippi State University. The samples, referred to as pseudo-ash, were manufactured by milling and sieving pre-existing volcanic deposits from five different locations. Magmas that produced these deposits have contrasting compositions, as two are derived from Strombolian eruptions of a basalt magma, three are derived from explosive eruptions of andesitic stratovolcanoes, and two are derived from explosive and effusive eruptions of a rhyolite magma. LW-ONW and LW-NINW were produced from scoria lapilli erupted during Strombolian explosions from Lathrop Wells, Nevada, 77 ka^44,45^. LW-ONW is scoria with obvious surface oxidation of the pyroclasts, while LW-NINW displays no evidence of surface oxidation. R-1 was produced from pumice lapilli erupted from Mt. Ruapehu, New Zealand during the Mangatoetoenui eruption ~20 ka^46^. K38-PC2 was prepared from pumice lapilli erupted from Gunung Kelud (East Java, Indonesia) during the February 2014 Plinian eruption^47,48^. SHV347-B was a pumice airfall pyroclast resulting from a Vulcanian explosion during the February 11, 2010 partial dome collapse at the Soufrière Hills volcano on the island of Montserrat in the British West Indies^49^. Due to the age of the R-1 sample, it displayed evidence of weathering and post-depositional alteration prior to collection. The other two intermediate samples were collected immediately after eruption and did not undergo any post-depositional alteration, and are thus considered ‘pristine’. OBS1 was derived from the Banco Bonito obsidian lava flow erupted from the Valles Caldera, New Mexico ~70 ka^50^, containing no observable crystals and very few spherulites. ECF2LAP was manufactured from crystal-poor pumice lapilli derived from the El Cajete series, also from the Valles Caldera, which was the last major explosive event from this volcano ~75 ka^50^. Both acidic samples showed no evidence of weathering or devitrification.

All samples were ground into very fine ash-sized (<32 μm) fragments using a McCrone micronising mill and sieved to ensure grain size homogeneity. Bulk compositions of the pre-experimental samples were measured with the Philips Analytical PW2400 X-ray fluorescence (XRF) analyzer to determine the relative proportions of major and minor oxide compounds. Mineral contents were measured with the Bruker AXS D8 X-ray diffractometer (XRD). Pre-experimental samples were also used to prepare polished epoxy mounts for scanning electron microscopic (SEM) analyses. Backscattered electron images of the samples were obtained with a JOEL JSM 6010 Plus/LA SEM operating at an accelerating voltage of 15–20 kV. Vibrating sample magnetometry (VSM: MicroSense Model-EV) was used to measure the saturation magnetization (*M*_s_), the remanent magnetization (*M*_r_), and the coercivity (*H*_c_) of the pre-experimental pseudo-ash.

Pseudo-ash samples (1–2 grams) were sprinkled on an aluminum alloy plate and subjected to a current impulse with a peak value of either ~100 kA, ~25 kA, or ~7 kA following previously described methods^2^. To insure arcs traveled through the sample, a steel screw was inserted into the plate above the sample mounts to provide a path for the current to ground (Fig. 1). Following the impulse experiments, samples were brushed from the aluminum plate and separated into different portions for post-experimental analyses. All portions of the subjected samples were collected simultaneously, so variations as a function of the position on the plate, and distance from the arc channel axis, cannot be determined. Thus, all measurements are an average for the bulk sample. Grain mounts were prepared by sprinkling portions of the post-experimental samples on carbon tape affixed to a 10 mm aluminum stub. These samples were used to obtain secondary electron images with the SEM. Polished epoxy mounts were also prepared for acquisition of backscattered electron images to examine impulse-induced textural changes in the pseudo-ash. VSM was again used to examine the magnetic properties of the post-experimental samples and compare to the pre-experimental values of *M*_*s*_, *M*_*r*_, and *H*_*c*_. All stages of sample preparation and analysis can be found in Fig. S15.

## Supplementary information

Supplementary Information

## Data Availability

The data discussed in this paper is presented in full in the figures, tables, and supplementary information.
