# Supplementary material for: Effects of Lightning on the Magnetic Properties of Volcanic Ash
Source: Sci Rep. 2019 Mar 18;9:4726. doi: 10.1038/s41598-019-41265-3 (PMC6423021; doi:10.1038/s41598-019-41265-3)
Supplement: Supplementary file 1 — Supplementary Information [file 41598_2019_41265_MOESM1_ESM.pdf]

Supplementary Information

Effects of Lightning on the Magnetic Properties of Volcanic Ash

Kimberly Genareau[1*], Yang-Ki Hong[2], Woncheol Lee[2], Minyeong Choi[2], Mojtaba Rostaghi-Chalaki[3], Pedram Gharghabi[3], James Gafford[4,5], and Joni Klüss[3]

[1]Department of Geological Sciences, The University of Alabama, Tuscaloosa, Alabama 35487, USA.

*kdg@ua.edu

[2]Department of Electrical and Computer Engineering, The University of Alabama, Tuscaloosa, Alabama 35487, USA.

[3]Department of Electrical and Computer Engineering, Mississippi State University, Starkville, Mississippi, 39762, USA.

[4]Center for Advanced Vehicular Systems at Mississippi State University, Starkville, Mississippi, 39759, USA.

[5]Present address: Energy Production and Infrastructure Center, The William States Lee College of Engineering, University of North Carolina, 9201 University City Blvd, Charlotte, North Carolina 28223, USA.

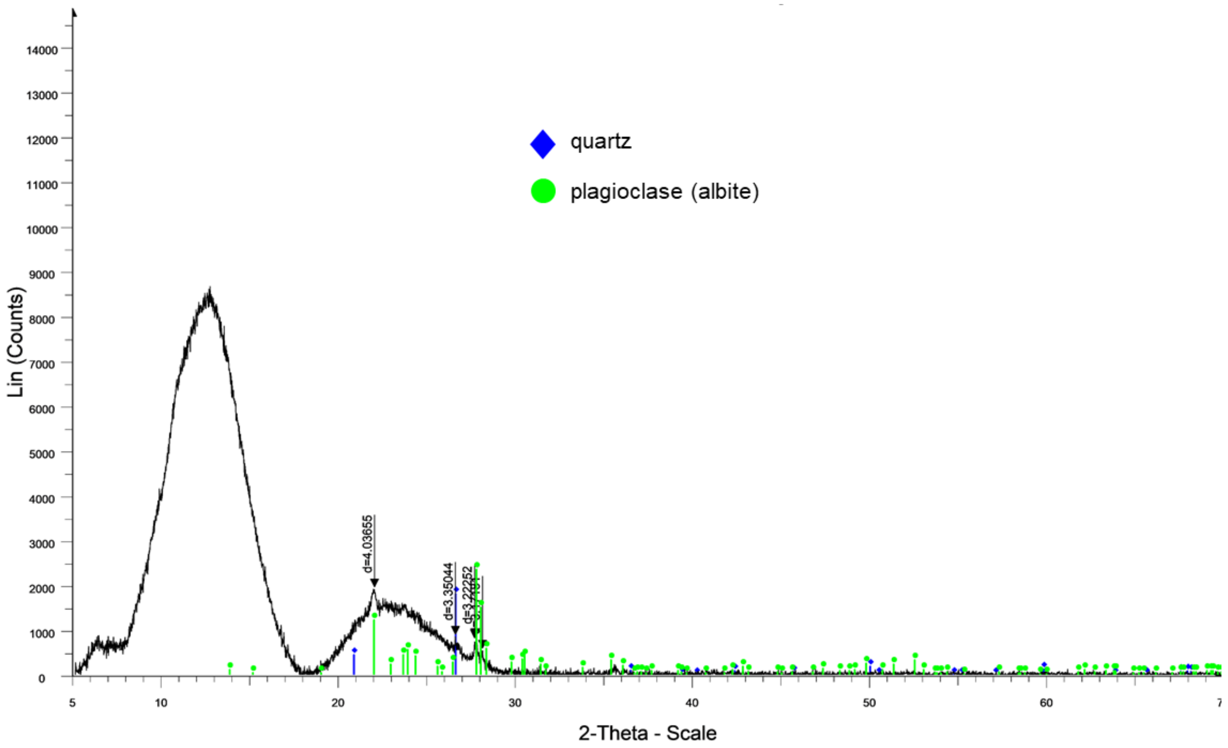

Figure S1. X-Ray diffraction spectrum of pre-experimental pseudo-ash sample OBS1. The high peak to the left indicates the glass component and the low, broad hump between 20° and 30° 2θ is amorphous silica.

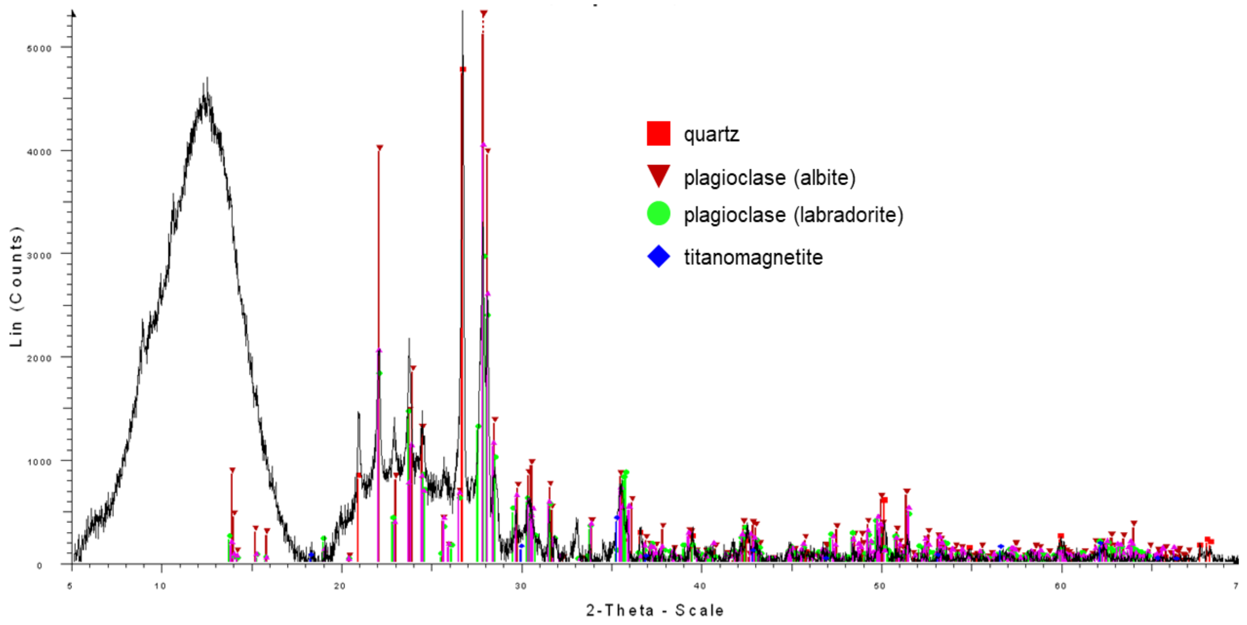

Figure S2. X-Ray diffraction spectrum of pre-experimental pseudo-ash sample ECF2LAP. The high peak to the left indicates the glass component and the low, broad hump between 20° and 30° 2θ is amorphous silica.

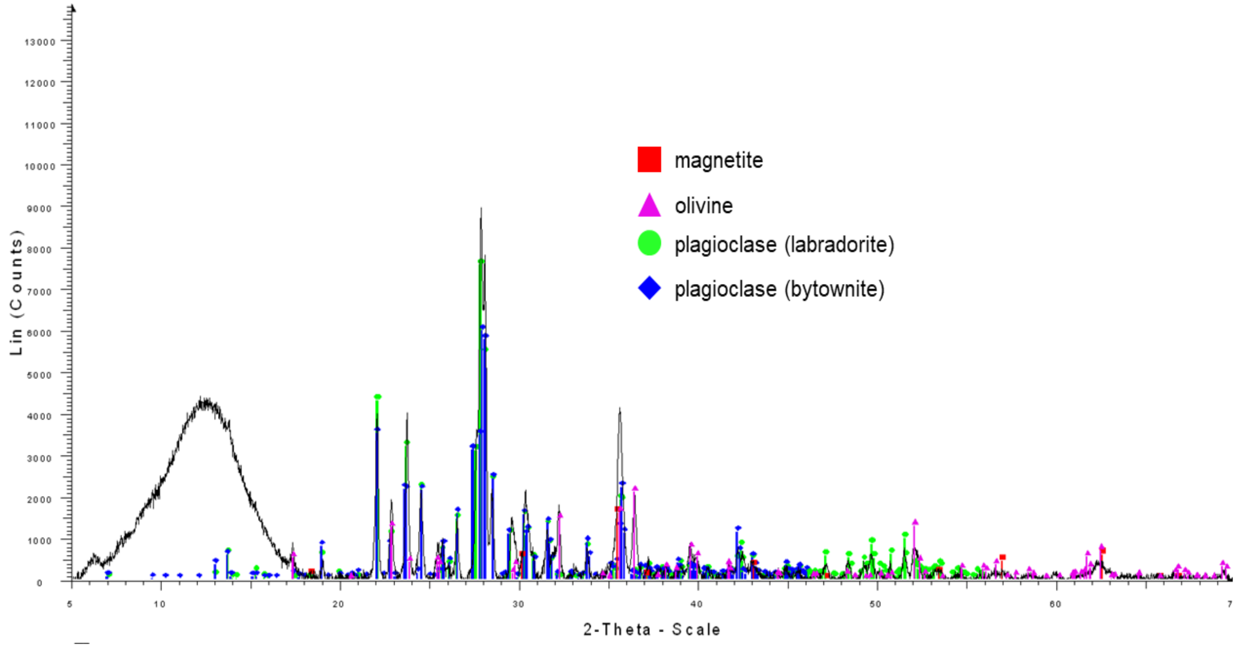

Figure S3. X-Ray diffraction spectrum of pre-experimental pseudo-ash sample LW-ONW. The peak to the left indicates the glass component.

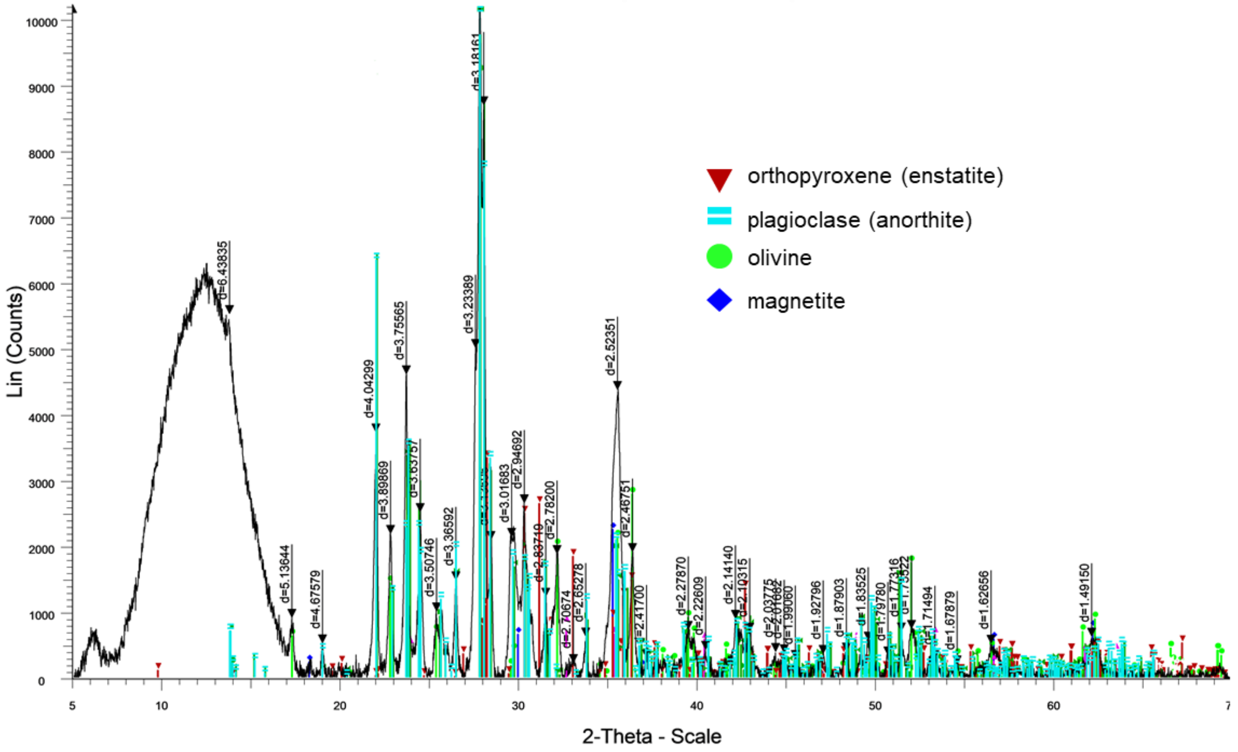

Figure S4. X-Ray diffraction spectrum of pre-experimental pseudo-ash sample LW-NINW. The high peak to the left indicates the glass component.

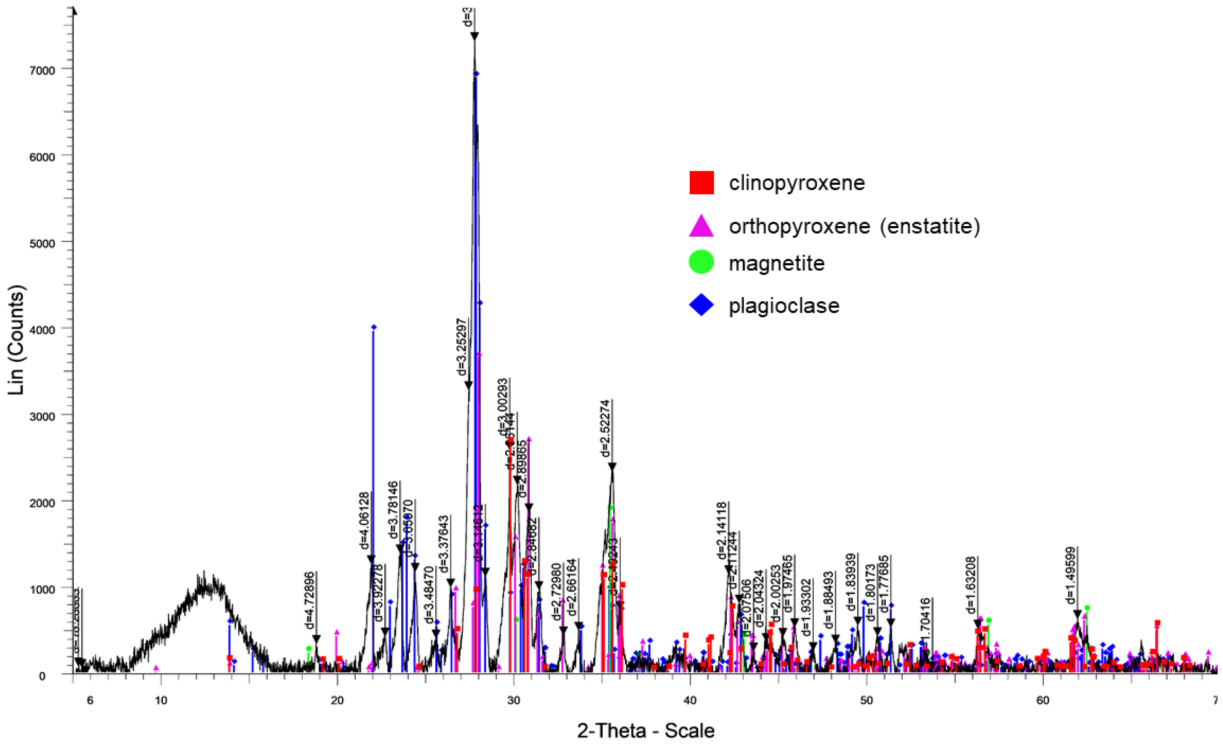

Figure S5. X-Ray diffraction spectrum of pre-experimental pseudo-ash sample R-1. The small peak to the left indicates the glass component.

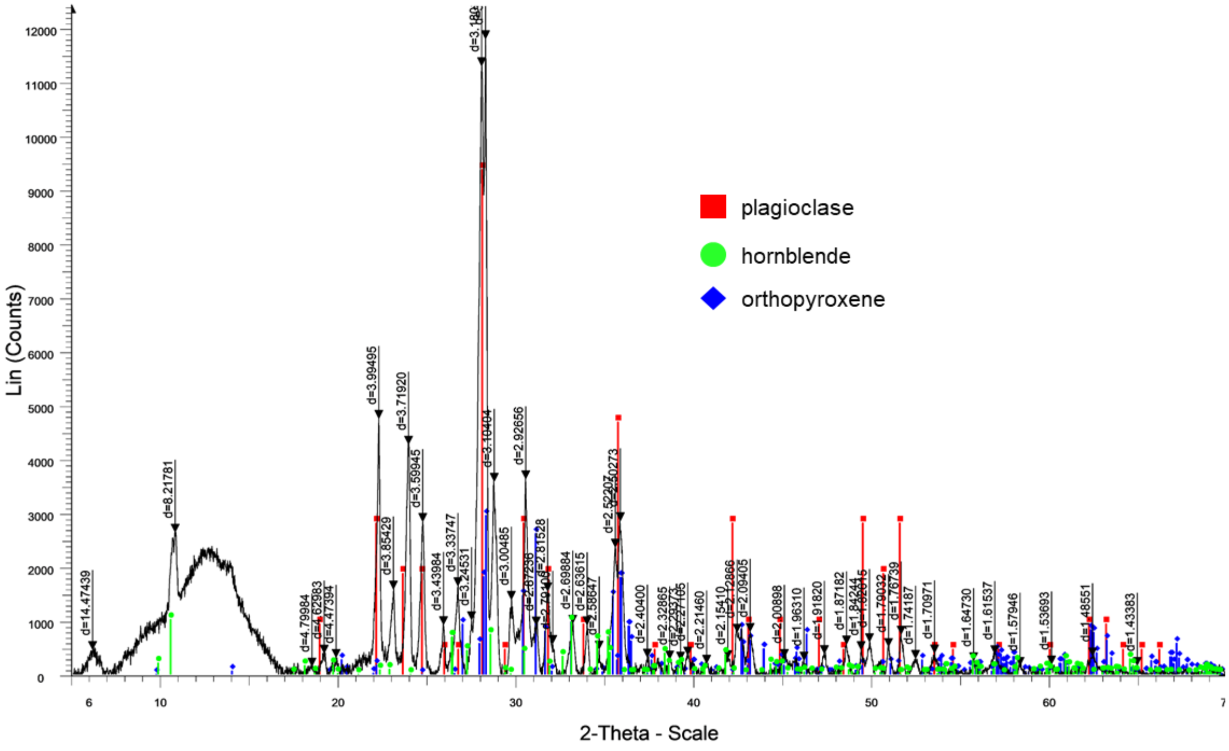

Figure S6. X-Ray diffraction spectrum of pre-experimental pseudo-ash sample SHV347-B. The small peak to the left indicates the glass component.

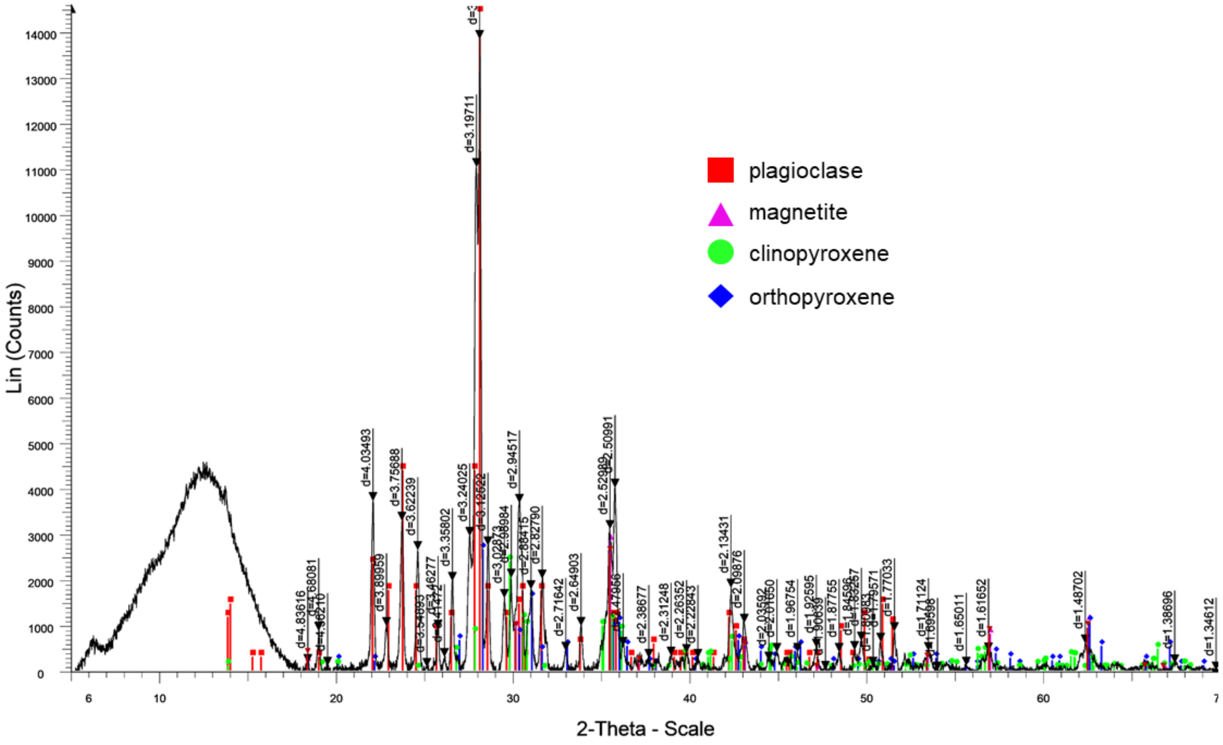

Figure S7. X-Ray diffraction spectrum of pre-experimental pseudo-ash sample K38-PC2. The peak to the left indicates the glass component.

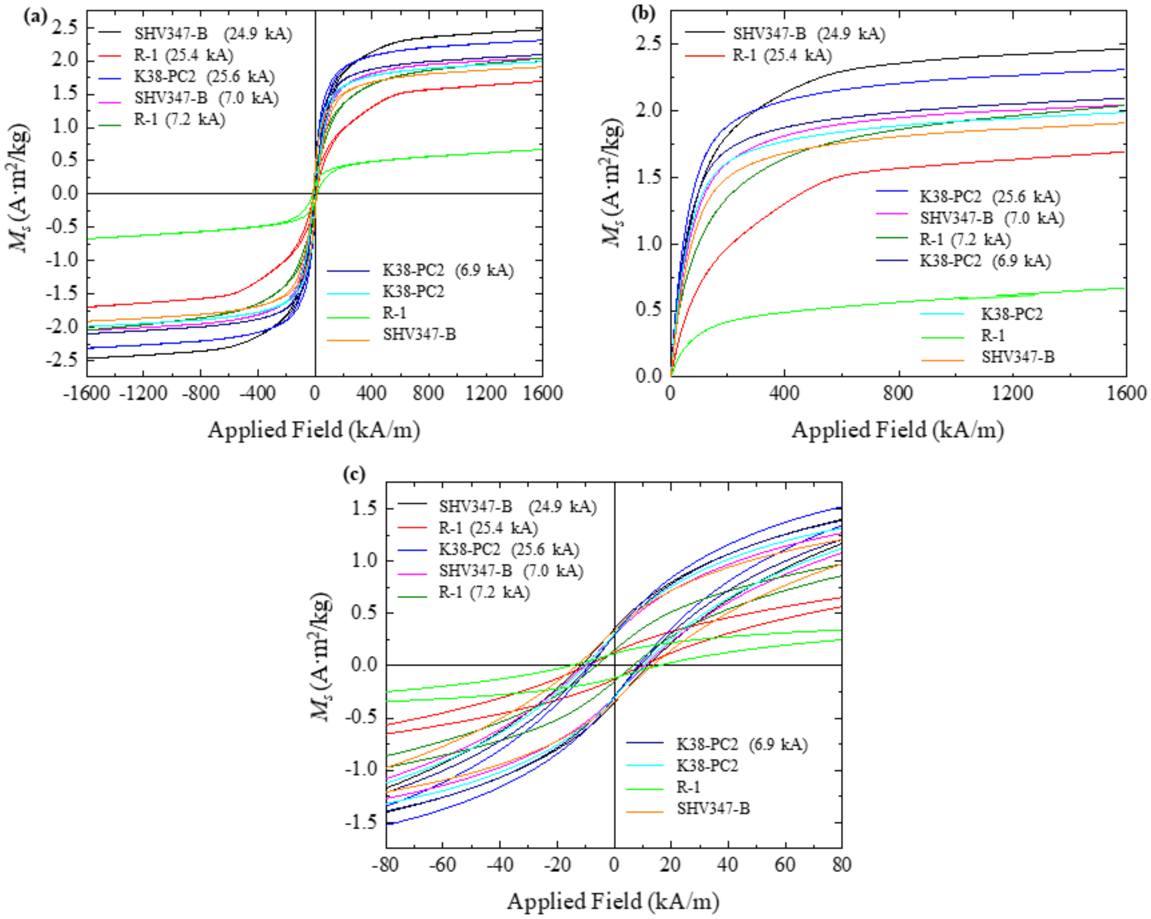

Figure S8. Hysteresis loops for variations in magnetization (M_S) between pre-experimental and post-experimental intermediate samples as a function of applied field. These samples represent a second set tested to check for instrument consistency and sample variability. Pre-experimental hysteresis loops are labeled with the name of the sample, while post-experimental loops are labeled with the sample name followed by the peak current value in parentheses. Pristine samples (K38-PC2 and SHV347-B) have higher magnetization values than sample R-1, which is much older and was weathered and oxidized prior to collection. **(a)** The entire loop under an applied field of -1600 to 1600 kA/m; **(b)** the positive quadrant under an applied field of 0 to 1600 kA/m; **(c)** the entire loop under an applied field of -80 to 80 kA/m. Error is within the thickness of the curve.

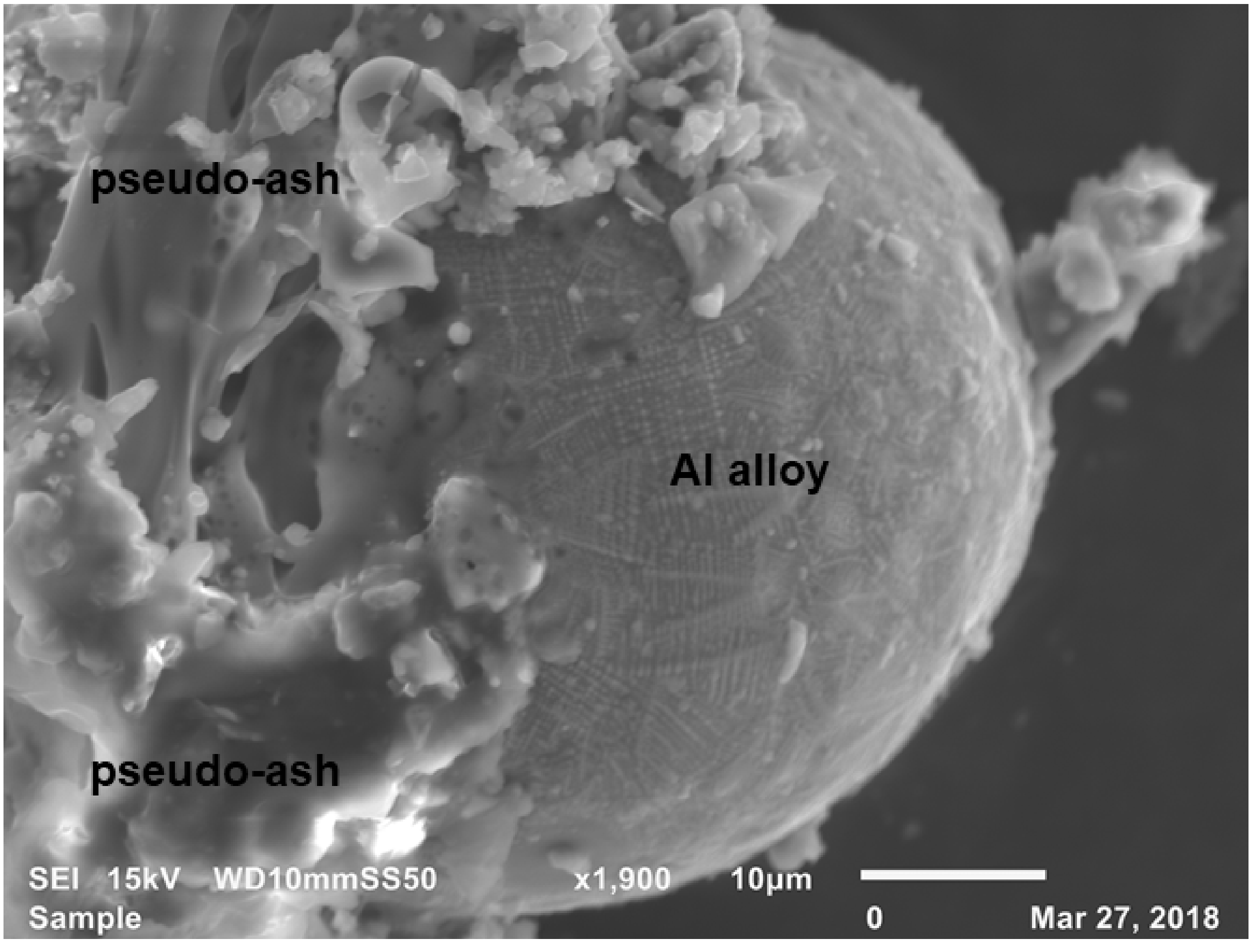

Figure S9. Secondary electron image of aluminum alloy spherule in post-experimental pseudo-ash sample. Less than five of these Al spherules were observed in the total set of post-experimental samples, and they were only observed in samples subjected to a current impulse of ~100 kA.

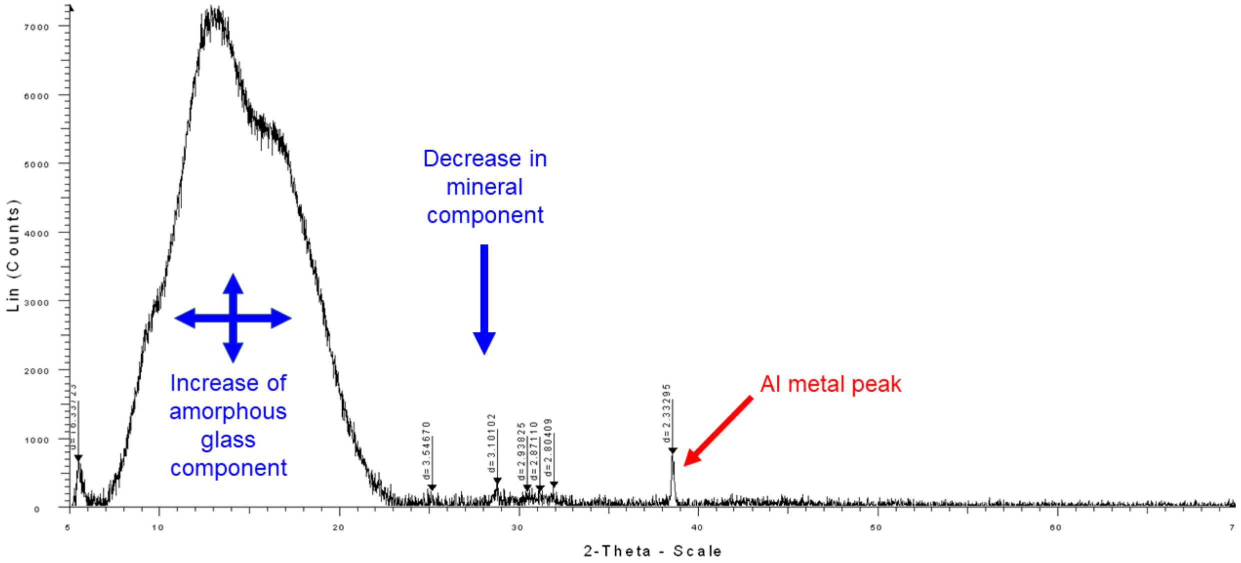

Figure S10. X-Ray diffraction spectrum of post-experimental pseudo-ash sample OBS1. Spectrum shows a decrease in mineral abundance compared to pre-experimental samples, although this reduction is partially due to the limited amount of post-experimental sample available for analysis. There is also an increase in both the height and width of the amorphous peak, indicating an increase in the glass component compared to pre-experimental samples. Only this sample revealed contamination from the Al alloy plate.

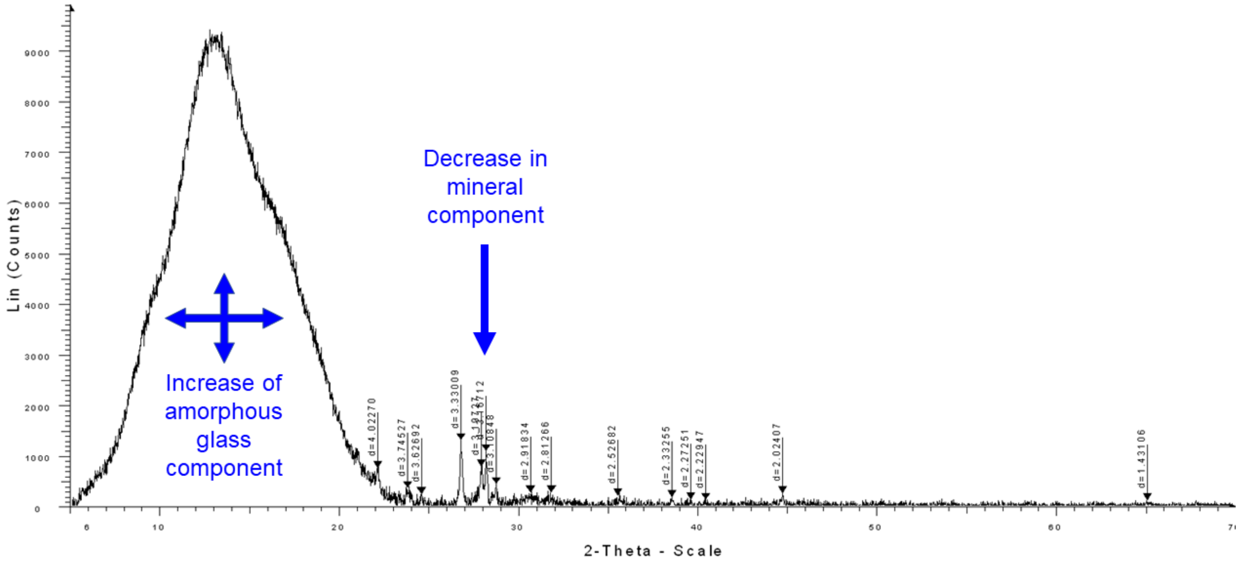

Figure S11. X-Ray diffraction spectrum of post-experimental pseudo-ash sample ECF2LAP. Spectrum shows a decrease in mineral abundance compared to pre-experimental samples, although this reduction is partially due to the limited amount of post-experimental sample available for analysis. There is also an increase in both the height and width of the amorphous peak, indicating an increase in the glass component compared to pre-experimental samples.

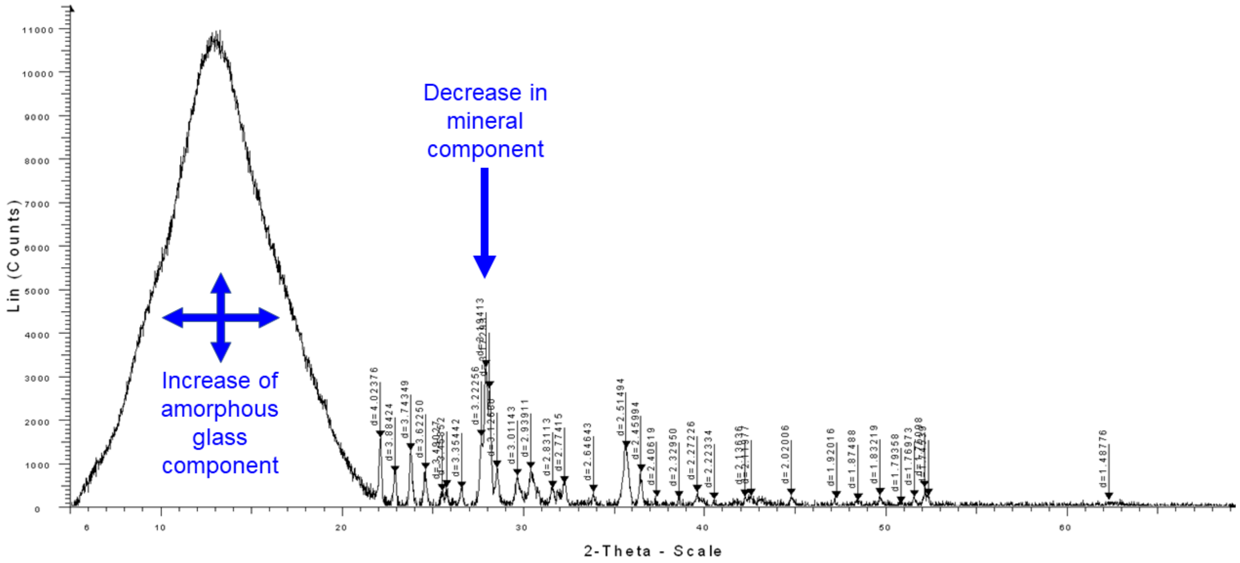

Figure S12. X-Ray diffraction spectrum of post-experimental pseudo-ash sample LW-ONW. Spectrum shows a decrease in mineral abundance compared to pre-experimental samples, although this reduction is partially due to the limited amount of post-experimental sample available for analysis. There is also an increase in both the height and width of the amorphous peak, indicating an increase in the glass component compared to pre-experimental samples.

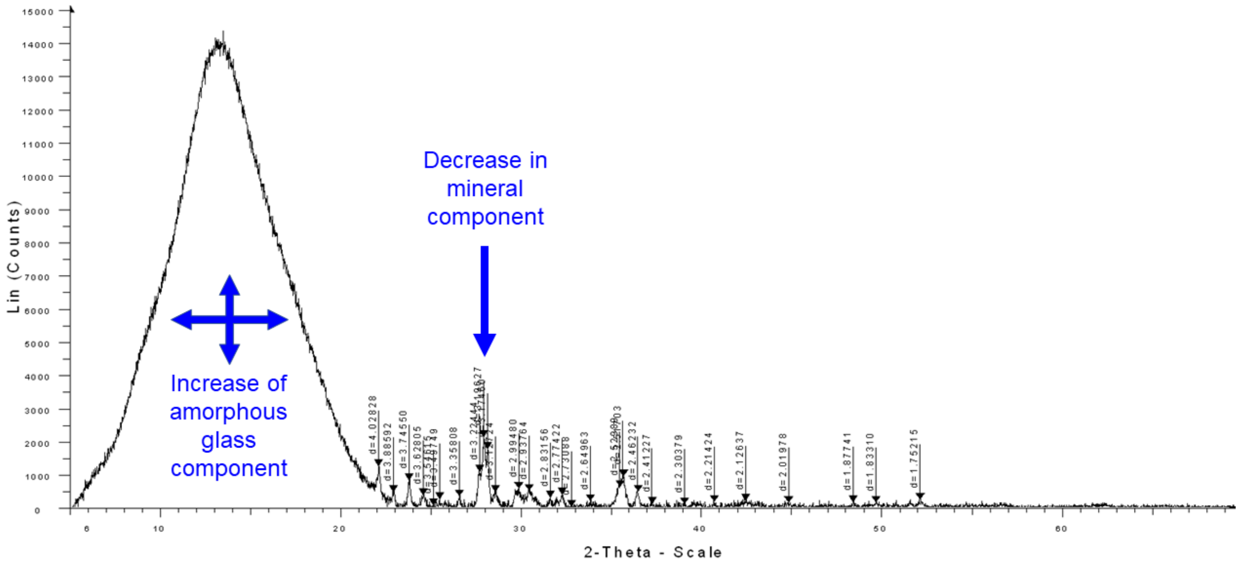

Figure S13. X-Ray diffraction spectrum of post-experimental pseudo-ash sample LW-NINW. Spectrum shows a decrease in mineral abundance compared to pre-experimental samples, although this reduction is partially due to the limited amount of post-experimental sample available for analysis. There is also an increase in both the height and width of the amorphous peak, indicating an increase in the glass component compared to pre-experimental samples.

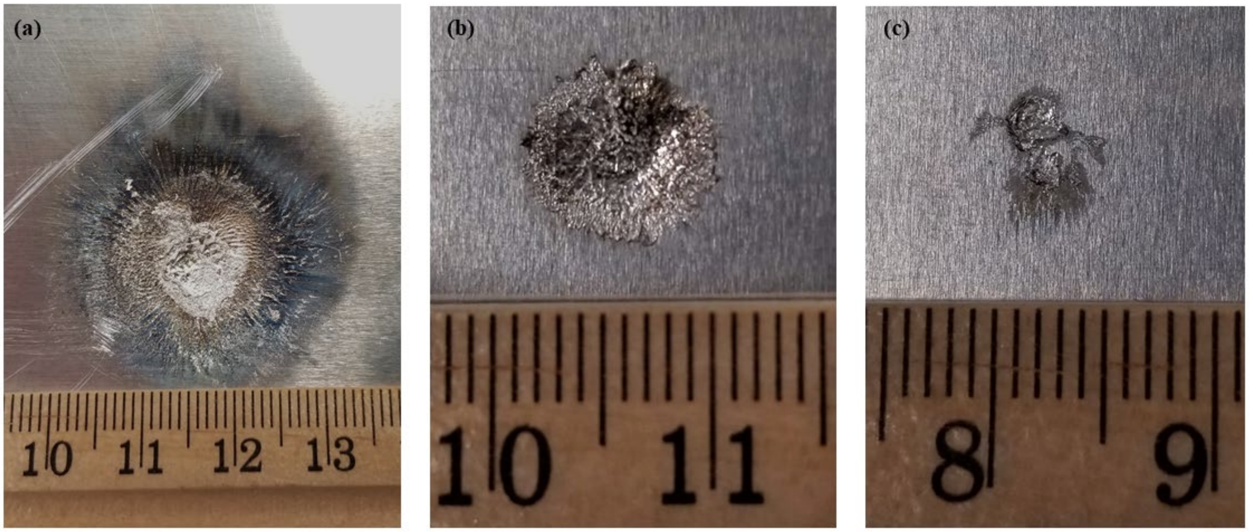

Figure S14. Photographs of post-experimental aluminum alloy plates. Diameter of the arc channel is indicated by the region of plate deformation. **(a)** ~100 kA current impulse; **(b)** ~25 kA current impulse; **(c)** ~7 kA current impulse. Only the 100 kA experiments showed evidence of Al oxidation and formation of Al spherules in post-experimental samples. Scale at the bottom of the images is in cm.

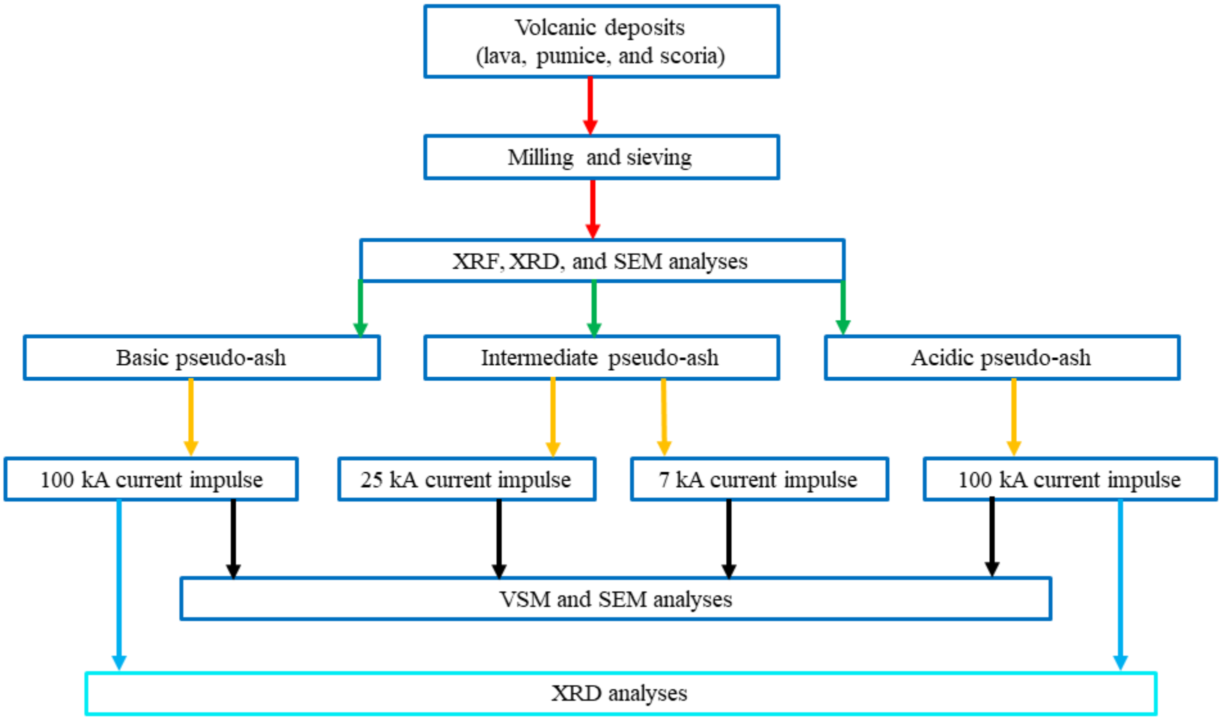

Figure S15. Block diagram showing various stages of pseudo-ash preparation and analysis. XRF = X-ray fluorescence; SEM = scanning electron microscopy; XRD = X-ray diffraction; VSM = vibrating sample magnetometry.
